# Sex Differences in Anxiety and Depression Among Coronary Heart Disease Patients During Cardiac Rehabilitation: A Quasi-Experimental Study

**DOI:** 10.3390/epidemiologia6030045

**Published:** 2025-08-07

**Authors:** Janne Grete Myklebust, Lotte Pannekoeke, Jon Arne Sandmæl, Harald Kåre Engan, Irene Lie, Christine Tørris

**Affiliations:** 1 Department of Nursing and Health Promotion, Oslo Metropolitan University, 0130 Oslo, Norway; jamykl@ous-hf.no (J.G.M.); lotte.pannekoeke@hvl.no (L.P.); 2 Center for Patient Centered Heart and Lung Research, Department of Cardiothoracic Surgery, Oslo University Hospital, 0424 Oslo, Norway; irene.lie@ous-hf.no; 3 Department of Health and Caring Sciences, Western Norway University of Applied Sciences, 5528 Haugesund, Norway; 4 Unicare Rehabilitation Norway, 0609 Oslo, Norway; jon.arne.sandmael@unicare.no (J.A.S.); harald.kare.engan@unicare.no (H.K.E.)

**Keywords:** anxiety, depression, coronary heart disease, cardiac rehabilitation, sex differences, women’s health

## Abstract

**Background/Objectives**: Anxiety and depression are common among individuals with coronary heart disease (CHD) and pose significant barriers to lifestyle modifications. Evidence on sex-related differences in anxiety and depression following cardiac rehabilitation (CR) remains inconclusive. This study aims to assesses the prevalence and changes in anxiety and depression symptoms during CR and explores potential sex differences. **Methods**: A quasi-experimental one-group pretest–post-test design was employed, measuring self-reported anxiety and depression symptoms utilizing the Hospital Anxiety and Depression Scale (HADS). **Results**: HADS was reported by 175 patients, 122 men and 53 women, at CR admission and discharge between 1 January 2022 and 30 April 2024. The prevalence of anxiety symptoms (HADS-anxiety score ≥ 8) significantly decreased from 28.2% at admission to 16.9% at discharge, while depression prevalence dropped (HADS-depression score ≥ 8) from 16.3% to 6.2%. Statistically significant sex differences were observed in depression prevalence at discharge, with women exhibiting lower symptom prevalence. Both sexes experienced significant HADS-anxiety and HADS-depression score reductions (*p* < 0.001) in both the overall sample and the sub-analysis of patients presenting with symptoms at admission. Women initially presented higher HADS-anxiety scores and significantly greater HADS-anxiety score reductions (*p* = 0.014) than men. No significant sex differences were observed in the reduction in HADS-depression scores. **Conclusions**: The prevalence of anxiety and depression symptoms significantly decreased among both sexes compared to admission, with women experiencing greater symptom reduction at discharge than men. Further research is needed to determine specific CR components contributing to these improvements.

## 1. Introduction

Cardiovascular diseases (CVDs) persist globally as leading causes of disability and mortality [1]. In Europe, CVDs account for 40% of all deaths in women and 35% in men, making it the primary cause of mortality [2]. Within the CVD spectrum, coronary heart diseases (CHDs) are the predominant contributors to morbidity and mortality [1,3,4]. CHDs are defined as diseases of the coronary arteries primarily associated with atherosclerosis leading to ischemia or myocardial infarction (MI) [1,5]. Sex differences in CHD diagnosis, treatment, and prognosis are undeniable and often unfavorable for women, resulting in a greater risk of poorer outcomes [6,7,8]. This disparity is compounded by a lack of awareness and knowledge among healthcare professionals, exacerbating sex inequality in cardiovascular health [6,7,8].

Anxiety and depression are highly prevalent and linked to poorer prognosis in CHD patients [9,10,11,12,13]. Moreover, anxiety and depression present significant barriers to lifestyle modifications, cardiac rehabilitation (CR) participation, adherence, and completion [2,14,15,16,17,18]. A Swiss pre–post design study (*n* = 3434) revealed that depression negatively impacts improvements in exercise capacity and quality of life in comprehensive CR [19]. Globally, the prevalence of anxiety and depression in cardiac patients is estimated to be 32.9% and 31.3%, respectively [20]. Among European CHD patients (*n* = 7589), 26.3% reported anxiety symptoms, and 22.4% reported depression symptoms [21]. Recent Norwegian data (*n* = 10,245) indicate a 30–40% prevalence of anxiety and/or depression symptoms in MI patients [22]. A Norwegian cohort study including 775 patients reported a 27.1% prevalence of anxiety and a 19% prevalence of depression after percutaneous coronary intervention, with higher prevalence rates in CR participants compared to non-participants [23]. Compared with men, women with CHD exhibit a greater prevalence and more severe anxiety and depression symptoms compared to men [14,21,24,25]. Recent studies agree that women with CHD entering CR in general present with higher anxiety and/or depression symptoms levels than men do [26,27,28,29,30,31,32,33]. Despite the significant prevalence and adverse impact of these mental health conditions on cardiovascular health, and the European guidelines recommending screening and treatment, anxiety and depression in CHD patients are frequently underdiagnosed and undertreated [15,34]. Moreover, psychological health traditionally receives less attention than physical health [15,34]. Identifying and treating these mental health challenges is essential when aiming to improve patient’s prognosis [2,35].

**Figure 1:**
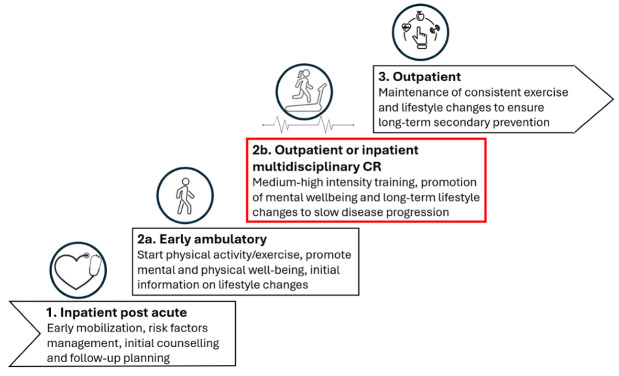
Phases in cardiac rehabilitation.

CR is a comprehensive, multidisciplinary intervention aimed at improving cardiovascular health and overall well-being [34]. The European Society of Cardiology (ESC) classifies CR as a class 1A recommendation and considers it a cost-effective intervention for secondary prevention through the assessment and treatment of cardiovascular risk factors [2]. In Norway, CR is offered in three phases, as recommended by ESC guidelines, each supporting patients at different recovery stages (Figure 1) [36,37]. Phase 2 CR is further divided into phases 2a and 2b, ensuring both early initiation and tailored multidisciplinary intervention [36]. Phase 2b typically involves outpatient group training or inpatient services at specialized institutions, which play a critical role in slowing disease progression and promoting sustainable lifestyle changes [36]. According to ESC guidelines, comprehensive CR should encompass assessment, physical exercise, lifestyle and risk factor management, education, counseling, and psychosocial support [2,34].

Exercise-only CR has been found to improve anxiety and depression symptoms in CHD patients [30,38]. Comprehensive CR, which synergistically integrates the advised CR elements, is however found to be most effective in facilitating positive patient outcomes; reducing CVD progression, readmission rates, and healthcare expenses, and promoting optimal physical and mental functioning for CHD patients [2,34,37,39,40,41,42]. Comprehensive CR provides a suitable context for anxiety and depression screening and effective treatment of these mental health issues, contributing to symptom reduction and consequently improving patients’ quality of life and prognosis [24,34,43]. While psychological interventions are found to improve depression and anxiety symptoms, uncertainty remains regarding the effect size and impact on adverse cardiac events and overall mortality of these additional psychosocial interventions to exercise-only CR [43,44,45]. Although both sexes may experience reduced anxiety and depression symptoms following CR completion, research studies are inconclusive regarding sex differences in the extent of these benefits [46]. While initial disparities in prevalence exist, with a higher prevalence in women than in men, sex differences in symptom reduction are not found in most studies following exercise-only CR [28,29,30] or comprehensive CR [26,27]. This finding might imply that once individuals are engaged in CR, the therapeutic benefits may be similarly effective across sexes [26,27,28,29,30]. However, the results are conflicting, with longitudinal studies indicating poorer symptom reduction in women at comprehensive CR completion [32,33].

To our knowledge, no Norwegian studies have examined the prevalence of anxiety and depression or changes in these symptoms during phase 2b CR, particularly with a focus on potential sex differences. The primary aim of this study is to examine the prevalence and changes in self-reported anxiety and depression symptoms among CHD patients completing phase 2b CR in Norway. Additionally, we aim to identify possible sex differences relating to anxiety and depression symptoms, accounting for possible sociodemographic and clinical confounders. Our research questions are as follows:

1. What is the prevalence of self-reported symptoms of anxiety and depression among CHD patients at admission and discharge from phase 2b CR, and are there sex differences?
2. How do self-reported anxiety and depression scores change from admission to discharge in CHD patients undergoing phase 2b CR, and are there sex differences?
3. Do CHD patients with anxiety and/or depression symptoms at CR admission report symptom reduction at discharge, and are there sex differences in this reduction?

## 2. Materials and Methods

### 2.1. Study Design, Setting and Intervention

This study used a retrospective pre–post quasi-experimental design. Data were collected for clinical treatment in a single phase 2b CR facility in central Norway. The data inclusion period spanned from 1 January 2022 to 30 April 2024. Patients were primarily admitted from The Central Norway Regional Health Authority. This CR facility offers a tailored inpatient program lasting three to four weeks, depending on the time of year (e.g., public holidays or seasonal breaks).

The program is designed based on an assessment of the patient’s functional and psychological status at admission. The primary objectives are to enhance functional ability, increase knowledge, and foster healthy behaviors. Additionally, psychosocial aspects regarding patients’ heart disease should be addressed. Ultimately, the program aims to motivate long-term self-care, improve coping mechanisms, manage symptoms, and reduce the risk of future cardiac issues. The comprehensive multifactorial program provides education, group guidance, individual support, medical evaluation, and daily physical training. Next of kin are involved and provided with guidance. A multidisciplinary team comprising nurses, physiotherapists, and a cardiologist are mainly responsible for coordinating patient care and managing treatment during the CR program. The physical training program includes a variety of resistance training and cardiovascular exercise, customized to individual needs and supervised by physiotherapists and/or nurses. When necessary, additional support is provided by an occupational therapist, psychiatric nurse, social worker, nutrition counsellor, or sports physiologist. Patients reporting anxiety and/or depression symptoms registered with the Hospital Anxiety and Depression Scale (HADS), with scores of ≥8, are considered to need further clinical interventions and receive additional supportive counseling during their stay, primarily from a psychiatric nurse. If necessary, they are also referred to municipal mental health services or specialized healthcare institutions for continued care.

### 2.2. Participants’ Characteristics, Data Collection, and Measures

Patients aged 18 years or older with a primary CHD diagnosis completing phase 2b CR were included in this study. For patients with multiple admissions during the data collection period, only data from the patient’s first stay were included to ensure that only one dataset was assessed per patient.

Sociodemographic data and information on anxiety and depression symptoms were collected primarily for clinical purposes via self-reported digital questionnaires featuring closed-ended questions. Data were collected at patient admission to CR (baseline) and at discharge (follow-up). The data were further stored in a registry database for research purposes. Additionally, details regarding the patient’s primary diagnosis, comorbidities, and medication regimen, sourced from medical records, were entered into the database. Primary diagnoses, as defined by the International Classification of Diseases (ICDs), were predominantly classified under atherosclerotic heart disease of native coronary artery (ICD-10-CM code I25.1), without further specification of CHD type or details regarding revascularization treatment. The included variables are presented in Table 1.

The Norwegian version of a validated generic patient-reported digital questionnaire, HADS, was used to screen CR participants for self-reported anxiety and depression symptoms at admission and discharge. The HADS is widely used in both national and international research across health care levels and patient groups to assess anxiety and/or depression symptoms [47]. The HADS comprises fourteen closed-ended questions, with seven addressing anxiety (HADS-A) and seven addressing depression (HADS-D). Each question offers four response alternatives, on a Likert-type summated rating scale from zero to three (the highest level of symptom burden) [47]. Summing the values from each question yields a total sub-score for HADS-A and HADS-D, ranging from zero to twenty-one [47]. This study applies an internationally recommended threshold value of ≥8, offering a good balance between sensitivity and specificity for the indication of anxiety (HADS-A) and depression (HADS-D) symptoms [47,48]. HADS has been shown to effectively measure psychological distress in CHD patients and has good internal consistency with the Cronbach’s alpha (HADS-A = 0.81–0.85, HADS-D = 0.78–0.88) [15,47].

### 2.3. Statistical Analysis

The included variables were assessed for normality. To describe the individual variables’ distributions, descriptive statistics and univariate analyses were utilized. The results are presented for each variable, with frequencies and percentages for categorical variables and medians and interquartile ranges (IQR) for continuous variables with skewed distributions [49,50]. Statistical significance for all analyses was determined at a *p*-value < 0.05.

Bivariate analyses were conducted to describe the empirical relationship between two variables and identify changes in HADS-A and HADS-D scores and sex differences. The results are presented in separate models for depression and anxiety.

To assess the possible influence of missing data on representativity, Pearson’s Chi-square Test for independence (categorical variables) and the Mann–Whitney U Test (nonparametric continuous variables) were used to evaluate differences between patients who did and did not report HADS [49].

To assess the prevalence rates of anxiety or depression symptoms, dichotomous HADS scores (≥8) were applied in univariate and bivariate analyses. McNemar’s test for repeated measures was applied to assess changes in the prevalence of anxiety and depression symptoms from admission to discharge [49].

Possible sex differences in categorical sociodemographic factors, clinical characteristics, and dichotomous HADS-A and HADS-D scores were assessed via Pearson’s Chi-square Test. Fisher’s exact test was used when Pearson’s Chi-square test assumption was violated with cell frequencies ≤5 [51]. The Mann–Whitney U test was used to assess sex differences in not normally distributed continuous variables, such as age, length of stay, and HADS score [49].

The Wilcoxon signed rank test was used for within-group comparisons to assess changes in HADS-A and HADS-D scores from admission to discharge for the whole sample, for participants reporting HADS scores ≥8 at admission, and for men and women in both groups [49].

Two multiple linear regression analyses were conducted to examine the relationships between the continuous dependent variable HADS-A or HADS-D at discharge and the independent variables HADS-A or HADS-D at admission and sex [49]. In addition, an interaction variable that combines sex and HADS-A score at admission or sex and HADS-D score at admission was included as an independent variable in the multiple linear regression analyses to identify whether differences between men and women were related to changes in anxiety/depression scores from admission to discharge [49]. Collinearity, multivariate outliers, normality, linearity, homoscedasticity, and independence of residuals in the multiple linear regression analyses were assessed [49]. Statistical analyses were carried out via IBM SPSS statistics, version 30.

### 2.4. Ethical Considerations

This study follows the ethical principles of the Helsinki declaration [52]. Approval was granted from the regional ethics committee for medical and healthcare research ethics (REK, 546776). All participants received comprehensive written information regarding the study’s objectives, procedures, and implications as part of an overarching research study completed at the same CR facility. Informed consent was obtained from each participant retrospectively by the institution responsible for data collection prior to the initiation of this study. The participants were explicitly informed of their right to withdraw consent. To safeguard participant anonymity and confidentiality, only deidentified data were utilized.

## 3. Results

### 3.1. Sample Characteristics

**Figure 2:**
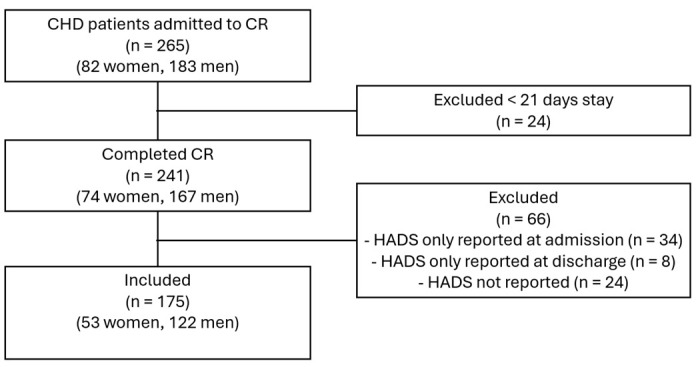
Flowchart of included and excluded patients.

From 1 January 2022, to 30 April 2024, 265 patients with a primary CHD diagnosis were admitted to the CR facility, and 241 of them completed three to four weeks of CR. Twenty-four patients, sixteen men and eight women with stays shorter than three weeks, were excluded, as they failed to complete the full CR program (Figure 2).

Among the 241 CR participants who completed CR, 175 (72.6%) reported HADS scores at both admission and discharge and were subsequently included in the analyses (Figure 2). Analyses of sociodemographic and clinical characteristics revealed that among patients with a complete dataset (*n* = 175) and those without (*n* = 66), the latter group had a greater proportion of retirees (*p* = 0.029) and previous cardiovascular events (*p* = 0.012). No other statistically significant differences were identified.

The sociodemographic and clinical characteristics of the participants included are detailed in Table 1. Women constituted roughly one-third of the sample. Participants’ median age was 66 years. A prior anxiety or depression diagnosis was found in 13.1% of the participants. Previous CHD and/or CVA/TIA were recorded for 22.3% of the participants, whereas 28.0% were diagnosed with diabetes prior to CR. Hypertension treatment was administered to 61.1% of the patients, while 95.4% were treated for hypercholesterolemia. A smoking history was reported in 30.3% of the patients, although only 9.1% were active smokers upon admission.

The Mann–Whitney U test for nonnormally distributed continuous variables and Pearson’s Chi-square test for categorical variables did not reveal statistically significant differences between men and women regarding sociodemographic factors and clinical characteristics (Table 1).

### 3.2. Prevalence of Self-Reported Anxiety and Depression

At admission, 28.2% of the participants exhibited anxiety symptoms (HADS-A ≥ 8) which were statistically significant (*p* < 0.001) and reduced to 16.9% by discharge. For depression (HADS-D ≥ 8), 16.3% of the participants reported symptoms at admission, statistically significant (*p* = 0.031) and decreasing to 6.2% at CR discharge. McNemar’s test for repeated measures revealed a statistically significant reduction in the prevalence of both anxiety (*p* = 0.003) and depression (*p* = 0.008) in women after CR. In contrast, men demonstrated a statistically significant reduction only in anxiety symptoms (*p* = 0.031), with no significant change in depression prevalence at CR completion.

Initially, women exhibited not statistically significant higher prevalence rates of anxiety and depression symptoms than the men. By discharge, both sexes had similar anxiety prevalence rates. Fisher’s exact test revealed a statistically significant association between sex and depression symptoms at discharge (*p* = 0.04), with women showing a lower prevalence of depression symptoms, indicating a greater reduction. However, the effect size was small (phi = −0.155).

### 3.3. Changes in Self-Reported Anxiety and Depression Scores

Table 2 provides an overview of the median (IQR) anxiety and depression scores reported by the patients at admission and discharge from CR, along with the changes in these scores from admission to discharge for the total sample, men and women. The range of HADS-A scores was 0–17 at admission and 0–18 at discharge, with median scores being reduced from 5 (IQR = 3–8) at admission to 4 (IQR = 2–6) at discharge. The HADS-D scores ranged from 0 to 16 at admission and 0 to 15 at discharge, with median scores of 4 (IQR = 2–7) at admission and 3 (IQR = 1–5) at discharge. The median changes in HADS-A and HADS-D scores were −1 (IQR = −3–0) and −1 (IQR = −2–0), respectively. The Wilcoxon signed rank tests indicated statistically significant anxiety (Z = −5.68, r = 0.30, *p* < 0.001) and depression (Z = −5.01, r = 0.27, *p* < 0.001) score reductions from admission to discharge.

The Mann–Whitney U Test, comparing HADS scores between sexes, revealed a statistically significant higher median HADS-A score (*p* = 0.006) in women than in men at admission. At discharge, no statistically significant difference between the sexes was identified. In terms of depression scores, men had higher median HADS-D scores than women did at both admission and discharge, although these differences were not statistically significant. A significant difference in the change in anxiety scores between sexes was identified (Z = −2.46, r = 0.3, *p* = 0.014), whereas no significant difference was found in the change in depression scores (Table 3).

The bivariate analysis did not reveal any differences between men and women in terms of sociodemographic factors or clinical characteristics that could confound sex differences in changes in HADS-A or HADS-D scores. Consequently, these factors were not included in the multiple linear regression analysis. The independent variables incorporated in the regression analyses were HADS scores at admission, sex, and the interaction variable sex × HADS.

The model summaries of the regression analyses indicate that the models accounted for 51.4% (R-square = 0.514) of the variance in the dependent variable HADS-A at discharge and 55.3% (R-square = 0.553) of the variance in the dependent variable HADS-D at discharge (Table 4). For both models, only HADS at admission significantly contributed to the model for HADS at discharge (*p* < 0.001). Specifically, HADS-A score at admission was positively correlated with HADS-A score at discharge, with regression coefficient B indicating that a one-unit increase in HADS-A score at admission was associated with an increase in HADS-A score at discharge of 0.65, controlling for other variables in the model. Similarly, HADS-D scores at admission and discharge were positively correlated with a one-unit increase in HADS-D scores at admission, corresponding to a 0.84 increase in HADS-D scores at discharge, when other variables were controlled for.

None of the other included independent variables contributed statistically to the variance in the dependent variable HADS-A/HADS-D in the multiple linear regression analyses. When adjusted for the baseline HADS-A/HADS-D score, no statistically significant sex differences related to the HADS-A or HADS-D score at discharge were observed. Furthermore, the interaction variable sex × HADS did not have a statistically significant effect on the variance in the dependent variables, indicating that the changes in HADS-A and HADS-D scores from admission to discharge were similar for both sexes.

### 3.4. Changes in HADS-Scores in Patients Presenting with Anxiety/Depression (HADS ≥ 8) at Admission

For patients reporting HADS scores exceeding the threshold value of 8 at admission (anxiety: *n* = 51, depression: *n* = 28), the changes in median scores were assessed via the Wilcoxon signed rank test for within-group comparisons. At discharge, the median HADS-A and HADS-D scores were reduced to below the threshold value. The median HADS-A score decreased from 10 (IQR = 9–11) at admission to 7 (IQR = 5–10) at discharge, whereas the median HADS-D score decreased from 9.5 (IQR = 8–11) to 7 (IQR = 5.25–9.75). The median reduction in HADS-A scores was −3 (IQR = −5–0) and −2.5 (IQR = −4–0.25) for HADS-D. These reductions were statistically significant for both the median HADS-A (Z = −4.664, *p* < 0.001) and HADS-D (Z = −3.770, *p* < 0.001) scores, with a medium effect size for changes in anxiety (r = 0.46) and a large effect size for changes in depression (r = 0.59) (Table 5).

Within this group, no statistically significant sex differences were found. Women reported a higher median HADS-A score than men did at admission, yet they had a lower median HADS-A score at discharge. The median HADS-D scores were higher for men than for women at both admission and discharge. Although not statistically significant, women in this patient group presented greater reductions in median anxiety and depression scores than men did (Table 6).

## 4. Discussion

### 4.1. Prevalence of Anxiety and Depression in CR

The proportion of patients reporting anxiety and depression symptoms in this study decreased significantly from admission to discharge, with rates decreasing from 28.2% to 16.9% for anxiety and from 16.3% to 6.2% for depression. These findings are, as expected, notably higher than the prevalence of anxiety and depression in the general population [53,54,55]. However, these figures are slightly lower than the global and European prevalence rates reported for CHD patients [20,21]. Similarly, the Norwegian MI Registry reported a higher prevalence, indicating that 30–40% of MI patients experience anxiety and/or depression symptoms [22]. Norwegian post-MI patients participating in CR exhibited a higher prevalence of anxiety (37.2%) and depression (27.3%) symptoms, which decreased to 18.8% and 14.1% three years later [23].

This study’s findings are consistent with international research evaluating anxiety and depression in patients completing phase 2 CR programs. For example, an Australian cohort study (*n* = 5908) reported anxiety and depression rates of 28% and 18%, respectively [16]. Similarly, a Swiss study reported an anxiety prevalence of 26.9% and a depression prevalence of 9.3%, with reductions of 24.3% and 5.0%, respectively, at comprehensive CR completion [19]. These findings suggest that phase 2 CR participants may present a lower prevalence of anxiety and depression symptoms than the broader CHD population does. Despite the strong recommendations and benefits of comprehensive CR [2,34], CHD patients with anxiety and depression symptoms might be more inclined to engage in alternative CR modalities, such as exercise-focused programs. ESC guidelines advocate for routine screening of anxiety and depression symptoms [2,34]. However, the implementation of such screening procedures remains inconsistent. Systematic screening should be integrated early in the disease trajectory to facilitate referrals to the most appropriate CR programs, thereby enhancing participation among patients with these psychological symptoms [56,57].

### 4.2. Changes in Anxiety and Depression Symptoms During CR

Previous research has demonstrated reduced depression and anxiety symptoms following exercise-only CR [24,29,30,31,46] and comprehensive CR [19,24,26,27,32,33]. Consistent with these findings, our study revealed statistically significant improvements in self-reported anxiety and depression symptoms after comprehensive CR. Specifically, the median HADS-A score decreased from 5 to 4, whereas the median HADS-D score decreased from 4 to 3. Patients presenting with anxiety and/or depression symptoms at admission (HADS ≥ 8) showed improvements, with median HADS-A scores significantly reduced from 10 to 7 and HADS-D scores from 9.5 to 7. Notably, both scores at discharge fell below the threshold value of 8, suggesting that on average, these patients no longer reported anxiety or depression symptoms upon completing CR. The medium and large effect sizes for reductions in HADS-A and HADS-D scores in this subgroup suggest that comprehensive CR may enhance mental health outcomes for the most affected patients.

The observed improvements are hypothesized to partly result from the synergistic effects of the core components of CR. However, this study did not isolate the contributions of individual components, leaving their specific impacts uncertain. Moreover, the optimal combination and delivery of CR components remain uncertain, as discussed in previous position papers [15,58]. The role of physical activity in improving mental health is well documented [59], and CHD patients experiencing anxiety and depression symptoms benefit from exercise interventions [38,39,60]. Recent systematic reviews have explored the additional impact of psychological interventions combined with exercise on CR, acknowledging their effectiveness in reducing anxiety and depression symptoms while expressing uncertainty regarding their influence on major adverse cardiac events and overall mortality [43,44,45]. Furthermore, considerable ambiguity persists concerning the intervention effect sizes and optimal delivery methods [43,44,45]. Educational strategies and social support may also play a vital role, as they have been found to positively influence patients’ mental health [15,58,61]. This calls for further research to elucidate the mechanisms underlying symptom reduction and to optimize CR delivery for enhanced mental health benefits.

### 4.3. Sex Differences Related to Anxiety and Depression

Although not significant, women in this study presented a greater prevalence of self-reported anxiety and depression symptoms than men did at admission to CR. Specifically, 37.7% of women reported anxiety and 25.5% of men reported anxiety, whereas depression was noted in 17% of women versus 15.6% of men. These disparities align with broader trends, indicating a generally higher prevalence of anxiety and depression among women [53,54,55]. Among European CHD patients, 39.4% of women and 22.1% of men experience anxiety, whereas depression affects 30.6% of women and 19.8% of men [21]. Additionally, anxiety symptoms in Norwegian CR were associated with being female [23]. Compared with the general European CHD population, the prevalence of depression symptoms was notably lower in women in this study, suggesting that women with depression might be insufficiently identified, referred, and admitted to comprehensive CR. As previous reviews have identified poorer psychosocial health in women as a barrier to CR participation, this should be further addressed [18,62].

HADS scores were significantly reduced for both sexes. At admission, women in this study had higher median HADS-A scores than men did; however, significantly greater reductions in HADS-A scores were observed at CR completion. No statistically significant sex differences were observed for changes in depression scores. Although not statistically significant, the women presenting with anxiety and/or depression at admission (HADS ≥ 8) reported greater reductions in median HADS scores than the men, suggesting that they might benefit slightly more. These observations are consistent with previous studies indicating that women present with higher levels of anxiety symptoms [16,19,26,27,28,29,30,31,32,33]. However, the findings for depression in this study contrast with those of previous studies indicating higher depression levels in women [16,19,26,27,28,29,30,31,32,33]. Conflicting evidence related to change has been reported in cohort studies, with no sex differences in depression reduction following exercise CR combined with education [27] or in anxiety and depression reduction at comprehensive CR completion [26]. A substantial US registry study (*n* = 27,670) reported less favorable outcomes in terms of depressive symptom reduction in women than in men after phase 2 CR [33]. Similarly, a Canadian cohort study (*n* = 278) indicated that women experienced fewer improvements in anxiety and depression symptoms than men did upon completing comprehensive outpatient CR [32].

An ESC position paper underscores the importance of tailored approaches to mental health challenges, emphasizing the need for CR programs to be adjusted to meet individual physical and psychosocial needs [15]. Counseling and enhancing social support might be particularly beneficial in improving women’s mental health in comprehensive CR [10,15,58,61]. Recent studies have suggested that women-focused or women-only CR might effectively address physical and mental health challenges [46,63]. However, the findings are inconclusive and both additional costs and practicalities should be considered [46,63,64,65]. A randomized controlled trial (*n* = 169) comparing the effects of home-based CR, mixed-sex CR, and women-only CR revealed no significant differences in health behaviors, psychosocial outcomes [65], program adherence, or functional capacity between the models [64]. A retrospective cohort study (*n* = 1181) further concluded that women completing CR alongside men achieved better physical outcomes than women did in women-only programs [66]. Thus, female-focused CR might not be essential when women are enrolled in comprehensive CR programs focused on tailoring interventions to patients’ individual needs but could contribute to increasing CR participation.

### 4.4. Sociodemographic and Clinical Characteristics

Although fewer women than men completed CR during the inclusion period, our study participants reflect the national distribution, as one in three patients diagnosed with CHD in Norway are women [67]. In contrast to previous research suggesting higher CR drop-out rates among women, particularly those with elevated depression and anxiety scores, this study’s drop-out rate was comparable between sexes [68,69]. This pattern aligns with previous Scandinavian findings, reporting fewer sex disparities in CR completion rates, possibly attributed to greater emphasis on sex equality in Scandinavia [70,71,72].

In terms of sociodemographic variables and clinical characteristics, no significant sex differences were observed among the participants in our study. Both men and women shared similar health profiles, including comorbidities, smoking status, and self-reported levels of anxiety and depression. The median age for men (66) and particularly women (65) in this study was noticeably lower than the median age for MI occurrence in Norway, 70 for men and 77 for women [22]. Additionally, only 9.1% of the participants in this study still smoked, whereas 23% of those in the general CHD population smoked. Further demographic analysis revealed that our study sample had a higher level of education than the general Norwegian population within the same age group [73]. Specifically, 36.1% of men and 41.5% of women in our study had completed higher education, in contrast to 29.4% of men and 36% of women in the general Norwegian population aged 60–66 years [73]. These findings are consistent with those of the NorStent study (*n* = 7068), which identified typical Norwegian CR participants as young and well educated [72]. Given that lower education levels are associated with higher MI rates due to poorer health behaviors and risk factor management, strengthening CR participation among less-educated individuals is crucial [74,75]. There is a need for targeted strategies to increase CR participation among demographics less represented in current programs, ensuring that CR health benefits are accessible to broader segments of the population.

### 4.5. Methodological Strengths and Limitations

This study’s practical application in clinical settings and ethical commitment to patient care are key strengths [76]. However, the quasi-experimental design without randomization and a control group limits internal validity, as it cannot isolate the effects of the rehabilitation program or account for natural improvements or other confounding factors [76]. The applied study design can only prove correlations between the studied variables but is not fit to establish causality [76]. Additionally, reliance on pre- and post-test data may affect accuracy due to participants’ familiarity with the questionnaire [77].

The use of a validated questionnaire is considered a strength [47,78]. However, the HADS is criticized for its nondiagnostic nature and is most effective at identifying overall distress or general psychological symptoms rather than serving as a diagnostic tool for anxiety and depression [47].

The small sample size, along with the limited number of women and cases meeting the HADS threshold, may obscure sex differences and constrain statistical analyses. Larger samples could enable parametric analyses and logistic regression [49].

This study’s external validity is limited due to the nonrepresentative sample of the general CHD population, as it includes only referred patients who agreed to participate and who completed phase 2b CR. The use of clinical data from a single site additionally reduces generalizability [76]. Patients with severe symptoms might have dropped out or not completed the HADS, potentially underestimating symptoms at admission and misinterpreting changes. However, the low drop-out rate (9.1%) and acceptable response rate (72.6%) are considered strengths of this study.

## 5. Conclusions

The prevalence of anxiety and depression symptoms significantly decreased among both sexes, with women experiencing greater symptom reduction at discharge than men. These findings align with international research, underscoring the potential of tailored comprehensive CR to address mental health challenges. However, further research is needed to identify the most effective CR components for optimizing patient outcomes.

## Figures and Tables

**Table 1:** Sociodemographic and clinical characteristics, anxiety and depression prevalence (*n* = 175), and sex differences.

Variable	Category	All		Men		Women		*p*-Value
		*n* = 175		*n* = 122 (69.7%)		*n* = 53 (30.3%)		
Sociodemographic factors								
Age-Median (IQR)	Years	66	59–71	66	58–71.25	65	59–71	0.915 ^a^
Length of stay-Median (IQR)	Days	28	22–28	28	22–28	28	22–28	0.511 ^a^
Educational level–*n* (%)	Primary	20	11.4	12	9.8	8	15.1	0.365 ^b^
	Secondary	89	50.9	66	54.1	23	43.4	
	Higher education	66	37.7	44	36.1	22	41.5	
Current occupation–*n* (%)	Employed (full-time or part-time)	67	38.3	48	39.3	19	35.8	0.708 ^b^
	Unemployed/benefits	55	31.4	36	29.5	19	35.8	
	Retired	53	30.3	38	31.1	15	28.3	
Children living at home–*n* (%)	Yes	21	12.0	12	9.8	9	17	0.279 ^b^
Living arrangement–*n* (%)	Living together with someone	127	72.6	93	76.2	34	64.2	0.144 ^b^
Clinical characteristics								
Medial History–*n* (%)	Previous CHD or CVA/TIA	39	22.3	32	26.2	7	13.2	0.088 ^b^
	Diabetes mellitus	49	28.0	34	27.9	15	28.3	1.0 ^b^
	Previous anxiety or depression diagnosis	23	13.1	12	9.8	11	20.8	0.085 ^b^
Treatment–*n* (%)	Hypertension treatment	107	61.1	73	59.8	34	64.2	0.712 ^b^
	Cholesterol treatment	167	95.4	118	96.7	49	92.5	0.396 ^b^
Smoking status–*n* (%)	Never	122	69.7	84	68.9	38	71.7	0.843 ^b^
	Former/current	53	30.3	38	31.1	15	28.3	
Self-reported anxiety and depression–dichotomous								
HADS-A admission–*n* (%)	≥8	51	29.10	31	25.4	20	37.7	0.148 ^b^
HADS-A discharge–*n* (%)	≥8	25	14.30	17	13.9	8	15.1	0.995 ^b^
HADS-D admission–*n* (%)	≥8	28	16	19	15.6	9	17	0.516 ^b^
HADS-D discharge–*n* (%)	≥8	10	5.80	9	7.4	1	1.9	0.04 * ^c^

^a^ Mann–Whitney U Test. ^b^ Pearsons’ Chi-Square Test. ^c^ Fisher’s Exact Test. * *p*-value < 0.05.

**Table 2:** Changes in HADS-A/HADS-D scores from admission to discharge in the sample (n = 175).

Variable		*n*	Admission		Discharge		Change		Z-Score	*p*-Value ^a^
HADS-A-Median (IQR)	All	175	5	3–8	4	2–6	−1	−3–0	−5.682	<0.001 *
	Men	122 (69.7%)	4.5	2–8	4	1.75–6	−0.5	−3–1	−3.662	<0.001 *
	Women	53 (30.3%)	6	4–9	5	2–6	−2	−4–0	−4.630	<0.001 *
HADS-D-Median (IQR)	All	175	4	2–7	3	1–5	−1	−2–0	−5.006	<0.001 *
	Men	122 (69.7%)	4	1–7	3	1–5.25	0.5	−2–1	−3.623	<0.001 *
	Women	53 (30.3%)	3	2–6	3	2–5	−1	−2.5–0	−3.650	<0.001 *

^a^ Wilcoxon signed rank test. * *p*-value < 0.05.

**Table 3:** Sex differences related to changes in HADS-A/HADS-D scores in the sample (*n* = 175).

Variable		All		Men		Women		Z-Score	*p*-Value ^a^
		*n* = 175		*n* = 122 (69.7%)		*n* = 53 (30.3%)			
HADS-A-Median (IQR)	Admission	5	3–8	4.5	2–8	6	4–9	2.744	0.006 *
	Discharge	4	2–6	4	1.75–6	5	2–6	1.084	0.278
	Change	−1	−3–0	−0.5	−3–1	−2	−4–0	−2.46	0.014 *
HADS-D-Median (IQR)	Admission	4	2–7	4	1–7	3	2–6	−0.253	0.8
	Discharge	3	1–5	3	1–5.25	3	2–5	−0.896	0.37
	Change	−1	−2–0	0.5	−2–1	−1	−2.5–0	−1.153	0.249

^a^ Mann–Whitney U test. * *p*-value < 0.05.

**Table 4:** Multiple linear regression analysis of the dependent variable HADS-A/HADS-D at discharge (*n* = 175).

Variable	Unstandardized Coefficients		Standardized Coefficients	T	Sig.	95% Confidence Interval for B	
	B	Std. Error	Beta			Lower Bound	Upper Bound
Model–dependent variable: HADS anxiety discharge							
(Constant)	1.572	1.068		1.472	0.143	−0.536	3.681
HADS-A admission	0.656	0.156	0.685	4.192	<0.001 *	0.347	0.964
Sex	−0.814	0.82	−0.106	−0.99	0.322	−2.432	0.805
Sex × HADS-A admission (interaction)	0.032	0.112	0.057	0.283	0.777	−0.19	0.253
Model–dependent variable: HADS depression discharge							
(Constant)	0.461	0.754		0.612	0.542	−1.027	1.95
HADS-D admission	0.84	0.138	0.952	6.098	<0.001 *	0.568	1.111
Sex	0.17	0.553	0.027	0.307	0.76	−0.922	1.261
Sex × HADS-D admission (interaction)	−0.154	0.104	−0.251	−1.49	0.139	−0.359	0.051

* *p*-value < 0.05.

**Table 5:** Changes in HADS-A/HADS-D scores from admission to discharge in patients with HADS ≥ 8 at admission.

Variable		*n*	Admission		Discharge		Change		Z-Score	*p*-Value ^a^
HADS-A- Median (IQR)	All	51	10	9–11	7	5–10	−3	−5–0	−4.664	<0.001 *
	Men	31 (60.8%)	10	9–11	8	5–10	−3	−4–0	−3.307	<0.001 *
	Women	20 (39.2%)	10.5	9–11.75	6.5	5.25–10	−4	−5–−0.25	−3.272	<0.001 *
HADS-D- Median (IQR)	All	28	9.5	8–11	7	5.25–9.75	−2.5	−4–−0.25	−3.770	<0.001 *
	Men	19 (64.7%)	10	9–12	7	6–11	−2	−3–0	−2.762	0.006 *
	Women	9 (35.3%)	9	8–10.5	7	4.5–7	−3	−5.5–−1.5	−2.524	0.012 *

^a^ Wilcoxon signed rank test. * *p*-value < 0.05.

**Table 6:** Sex differences related to changes in HADS-A/HADS-D scores in patients with HADS ≥ 8 at admission.

Variable		All		Men		Women		Z-Score	*p*-Value ^a^
		*n* = 51		*n* = 31 (60.8%)		*n* = 20 (39.2%)			
HADS-A-Median (IQR)	Admission	10	9–11	10	9–11	10.5	9–11.75	0.707	0.479
	Discharge	7	5–10	8	5–10	6.5	5.25–10	−0.418	0.676
	Change	−3	−5–0	−3	−4–0	−4	−5–−0.25	−0.903	0.366
		*n* = 28		*n* = 19 (64.7%)		*n* = 9 (35.3%)			
HADS-D-Median (IQR)	Admission	9.5	8–11	10	9–12	9	8–10.5	−1.207	0.243
	Discharge	7	5.25–9.75	7	6–11	7	4.5–7	−1.522	0.142
	Change	−2.5	−4–−0.25	−2	−3–0	−3	−5.5–−1.5	−1.316	0.205

^a^ Mann–Whitney U test. *p*-value < 0.05.

## Data Availability

The datasets generated during and analyzed during the current study are not publicly available due to restrictions in the participants’ signed consent.

## Abbreviations

The following abbreviations are used in this manuscript:

CHD	Coronary Heart Disease
CR	Cardiac Rehabilitation
CVA	Cerebrovascular Accident
ESC	European Society of Cardiology
HADS	Hospital Anxiety and Depression Scale
HADS-A	Hospital Anxiety and Depression Scale-Anxiety
HADS-D	Hospital Anxiety and Depression Scale-Depression
ICD	International Classification of Diseases and Related Health Problems
IQR	Interquartile Range
MI	Myocardial Infarction
TIA	Transient Ischemic Attack
