# Correction: Myklebust et al. Sex Differences in Anxiety and Depression Among Coronary Heart Disease Patients During Cardiac Rehabilitation: A Quasi-Experimental Study. *Epidemiologia* 2025, *6*, 45

**DOI:** 10.3390/epidemiologia7020031

**Published:** 2026-02-27

**Authors:** Janne Grete Myklebust, Lotte Pannekoeke, Jon Arne Sandmæl, Harald Kåre Engan, Irene Lie, Christine Tørris

**Affiliations:** 1 Department of Nursing and Health Promotion, Oslo Metropolitan University, 0130 Oslo, Norway; jamykl@ous-hf.no (J.G.M.); lotte.pannekoeke@hvl.no (L.P.); 2 Center for Patient Centered Heart and Lung Research, Department of Cardiothoracic Surgery, Oslo University Hospital, 0424 Oslo, Norway; irene.lie@ous-hf.no; 3 Department of Health and Caring Sciences, Western Norway University of Applied Sciences, 5528 Haugesund, Norway; 4 Unicare Rehabilitation Norway, 0609 Oslo, Norway; jon.arne.sandmael@unicare.no (J.A.S.); harald.kare.engan@unicare.no (H.K.E.)

Addition of Authors

Jon Arne Sandmæl and Harald Kåre Engan were not included as authors in the original publication [1]. The corrected Author Contributions statement appears here. 

Author Contributions: J.G.M. and L.P. are shared first authors, contributed equally, and wrote the main manuscript text together; J.G.M., L.P. and C.T. made substantial contributions to the analysis of the work and prepared the figures; J.G.M., L.P., I.L. and C.T. participated in the interpretation of the data; J.A.S. and H.K.E. contributed resources; All authors made substantial contributions to the conceptualization and design of the work and reviewed the manuscript. All authors have read and agreed to the published version of the manuscript.

Additional Affiliations

In the published publication, there was an error regarding the affiliations for Jon Arne Sandmæl and Harald Kåre Engan. In addition to affiliations 1, 2, 3, the updated affiliations should include: 4 Unicare Rehabilitation Norway, Oslo, Norway; jon.arne.sandmael@unicare.no (J.A.S.); harald.kare.engan@unicare.no (H.K.E.). 

The authors state that the scientific conclusions are unaffected. This correction was approved by the Academic Editor. The original publication has also been updated.

All authors have read and agreed to the published version of the manuscript.
